# BioGPS Descriptors for Rational Engineering of Enzyme Promiscuity and Structure Based Bioinformatic Analysis

**DOI:** 10.1371/journal.pone.0109354

**Published:** 2014-10-29

**Authors:** Valerio Ferrario, Lydia Siragusa, Cynthia Ebert, Massimo Baroni, Marco Foscato, Gabriele Cruciani, Lucia Gardossi

**Affiliations:** 1 Laboratory of Applied and Computational Biocatalysis, Dipartimento di Scienze Chimiche e Farmaceutiche, Università degli Studi di Trieste, Trieste (TS), Italy; 2 Laboratory for Chemometrics and Molecular Modeling, Dipartimento di Chimica, Biologia e Biotecnologie, Università degli Studi di Perugia, Perugia (PG), Italy; 3 Molecular Discovery Limited, Middlesex, London, United Kingdom; Instituto de Tecnologica Química e Biológica, UNL, Portugal

## Abstract

A new bioinformatic methodology was developed founded on the Unsupervised Pattern Cognition Analysis of GRID-based BioGPS descriptors (Global Positioning System in Biological Space). The procedure relies entirely on three-dimensional structure analysis of enzymes and does not stem from sequence or structure alignment. The BioGPS descriptors account for chemical, geometrical and physical-chemical features of enzymes and are able to describe comprehensively the active site of enzymes in terms of “pre-organized environment” able to stabilize the transition state of a given reaction. The efficiency of this new bioinformatic strategy was demonstrated by the consistent clustering of four different Ser hydrolases classes, which are characterized by the same active site organization but able to catalyze different reactions. The method was validated by considering, as a case study, the engineering of amidase activity into the scaffold of a lipase. The BioGPS tool predicted correctly the properties of lipase variants, as demonstrated by the projection of mutants inside the BioGPS “roadmap”.

## Introduction

Over the past decade, enzyme properties have been tailored both through evolutionary approaches as well as by applying computer-aided rational strategies [1]–[3]. Nevertheless, the recent scientific advances in computational research offer an array of tools that still have to express their full applicative potential. The huge amount of available information, provided by the revolution in life sciences, is far from being fully exploited. Although structures and sequences are expansively made available through databases, extracting complex information in a systematic way remains a difficult task: faster and more effective strategies are requested for discovering and developing new efficient enzymes for practical and industrial applications [4]. More specifically, the rational re-design of the active site of an enzyme necessitates of effective computational strategies able to evaluate how structural features are correlated to the ability of the protein to stabilize the transition state of a given reaction. Hybrid and comprehensive computational approaches are requested for exploring not only the structural complexity of enzymes but also for disclosing further factors that, by exerting their effect jointly, produce an optimized, pre-organized reaction environment. Aiming to meet these requirements, here we report on a novel computational methodology founded on the Unsupervised Pattern Cognition Analysis (UPCA) [4] of GRID-based BioGPS descriptors (Global Positioning System in Biological Space). The method aims to include in the analysis of enzyme active site the influence of physical-chemical factors that determine the “pre-organized reaction environment”, in analogy to the effect exerted by solvents on *in vitro* chemical reactions. Such analyses rely on the quantitative information extracted by the BioGPS molecular descriptors, calculated using the FLAP (Fingerprints for Ligands and Proteins) algorithm [5], which has been already used successfully in the field of drug design [6], [7]. The complexity of the information conveyed by descriptors is mastered by multivariate statistical analysis, more specifically by the application of Unsupervised Pattern Cognition Analysis. More importantly, the BioGPS-UPCA method relies neither on simple sequence alignment nor on structure superimposition but rather it involves the quantification of (macro)-molecular fingerprints, used to generate *de novo* virtual structures. On that basis, and with a modest computational cost, protein families or bunch of virtual mutants are compared and clustered overcoming any bias related to previous knowledge.

The present methodology intends to fill the existing gap in the field of computational methodologies, where the fundamental knowledge of the multiple structural and electronic factors cannot be correlated yet to catalytic properties, mostly because the effects of variables are studied separately. During the last decades Molecular Mechanics (MM) [8] and Quantum Mechanics (QM) have been used for designing novel structural features inside the protein scaffold [9]–[12] and the scientific literature reports some remarkable successful examples, although produced through the modification of a restricted number of structural or electronic features [13]. Studies based on QM theory level are also reported [14], where energies of the TS stabilized by the enzymes are calculated with high accuracy but accounting only for a limited section of the enzyme structure because of the high computational cost of the approach.

Besides MM and QM methods, different bioinformatic methodologies are also available but they compare mainly enzyme sequences [15]–[17], as, for instance, the ProSAR approach (Protein Sequence Activity Relationship) [18]. More in detail, the ProSAR approach analyses the sequence-function relationship of enzymes variants, taken from nature or generated in-vitro: the statistical weight of each residue is analyzed and this output is exploited for guiding the mutagenesis [18].

A more recent evolution of this approach is represented by the 3DM method, which allows retaining only part of the three-dimensional structural information. However the method requires a preliminary superimposition of structures so that afterwards residues are renumbered and sequences are compared on a different structural and conceptual basis [19].

In the present study, the efficacy of the BioGPS-UPCA method was tested by considering, as a case study, the engineering of amidase activity into the scaffold of lipase B from *Candida antarctica* (CaLB) [20], the aim was to engineer promiscuous amidase activity into the known and stable lipase scaffolds of CaLB, thus widening the array of applications of biocatalysts at industrial level.

The structure-based bioinformatic strategy started from the comparison of the active sites of 42 Ser-hydrolases belonging to four different classes: lipases, esterases, proteases and amidases. The 3D-structures of the active sites were subjected to BioGPS-UPCA procedure in order to understand why all enzymes of the serine-hydrolases super-family share, apparently, similar catalytic machineries but catalyze the hydrolysis of different chemical groups, namely esters and amides.

Unsupervised Pattern Cognition Analysis allowed for the unbiased identification and quantification of differences among hydrolases enzymes and, consequently, for their grouping inside clusters. The method was finally validated by projecting structures of CaLB mutants endowed with improved amidase activity into the Ser-hydrolases domain and by observing their clustering within the amidase area. Conversely, by analyzing and screening the virtual mutants, the method allowed for the *in-silico* monitoring of the effectiveness of a specific mutation strategy towards a desired engineering direction.

## Methods

### Definition of the serine-hydrolases data set

A dataset of 42 serine hydrolases was chosen in order to have a broad heterogeneity in terms of Ser hydrolases, also within the same class. Crystal structures of all the dataset enzymes were retrieved from the Protein Data Bank (PDB) [21] and preprocessed by using the software PyMOL [22]: all molecules but the proteins were deleted (i.e. water molecules, inhibitors, glycosylation residues, etc.). These 42 enzymes were separated into 4 enzyme classes considering their E.C. number: lipases (serine hydrolases defined as triacylglycerol lipase; E.C. 3.1.1.3), esterases (other carboxylic ester hydrolases but not triacylglycerol lipase; E.C. 3.1.1), proteases (serine endopeptidase; E.C. 3.4.21) and amidases (amino peptidase and other hydrolases acting on carbon-nitrogen bonds other than peptide bonds; E.C. 3.4.11, E.C. 3.5.1, E.C. 3.5.2).

### Active site superimposition for preliminary visual inspection

The preliminary visual inspection of the 42 Ser hydrolases was performed by superimposing their structures, although the results clearly show that a simple geometrical analysis is insufficient for identifying the critical structural differences. Because of the low homology inside the data set, structure superimposition was performed by taking the catalytic machineries as a reference point. Therefore, catalytic residues were aligned by superposing three functionalities of each enzyme: the atom acting as the general base during the catalysis (i.e. the Nε2 of His224 in CaLB) and the two mainly conserved H-bond donors constituting the oxyanion hole (i.e. the Nα atoms of Thr40 and Gln106 in CaLB). The residues considered for the superimposition and the relative catalytic Ser of each enzyme are indicated in Table S1. Afterward, the superimposition was performed by using an *ad hoc* Fortran script based on the Horn algorithm [23]. It performs iterative Horn superimposition, which runs with the objective of achieving a further refinement of the alignment. In the present case 15 iterative superimposition runs were performed. Due to the intrinsic stereochemistry of enzyme active sites, Ser hydrolases might have specular catalytic machineries making the superimposition impossible. Thus, in order to allow the catalytic machinery based superimposition, a geometrical reflection operation was performed when necessary by running an ad-hoc Python script that changes the sign of the first coordinate value of each atom of the structure, indeed, building up the enantiomeric enzyme structures; the reflected structures are indicated in Table 1.

**Table 1:** Serine hydrolases considered in this work with details on enzyme classes.

Enzyme class	PDB code	Source	Substrate
Lipases	1CRL	*Candida rugosa*	triacylglycerol
	1DTE	*Humicola lanuginosa*	triacylglycerol
	1ETH	*Sus scrofa*	triacylglycerol
	1EX9	*Pseudomonas aeruginosa*	triacylglycerol
	1GPL	*Cavia porcellus*	triacylglycerol
	1K8Q	*Canis lupus familiaris*	triacylglycerol
	1LPB	*Homo sapiens*	triacylglycerol
	1TCA	*Candida antarctica*	triacylglycerol
	2FX5	*Pseudomonas mendocina*	triacylglycerol
	2NW6	*Burkholderia cepacia*	triacylglycerol
	2W22	*Geobacillus thermocatenulatus*	triacylglycerol
Esterases	1AUO	*Pseudomonas fluorescens*	broad specificity
	1BS9	*Penicillium purpurogenum*	xylanes acetates
	^*^1C7J	*Bacillus subtilis*	p-nitrobenzyl esters
	1CLE	*Candida cylindracea*	cholesterol esters
	1JU3	*Rhodococcus sp.*	cocaine
	^*^1QOZ	*Tricoderma reesei*	xylanes acetates
	1USW	*Aspergillus niger*	feroloyl-polysaccharide
	2ACE	*Torpedo californica*	acetylcoline
	^*^2H7C	*Homo sapiens*	CoA, palmitate and taurocholate
	2WFL	*Rauvolfia serpentine*	polyneuridine aldehyde
	^*^3KVN	*Pseudomonas aeruginosa*	rhamnolipids
Proteases	^*^1GVK	*Sus scrofa*	Ala-|-Xaa
	^*^1NPM	*Mus musclus*	Lys/Arg-|-Xaa
	^*^1PPB	*Homo sapiens*	Arg-|-Gly fibrinogen
	1QFM	*Sus scrofa*	Pro-|-Xaa (∼30aa)
	^*^1TAW	*Bos Taurus*	Lys/Arg-|-Xaa
	^*^1TM1	*Bacillus amyloliquefaciens*	uncharged P1
	^*^1YU6	*Bacillus licheniformis*	uncharged P1
	2XE4	*Leshmania major*	olygopeptides
	^*^3F7O	*Peacelomyces lilacinus*	peptides
Amidases	1AZW	*Xantomonas campestris*	NH-Pro-|-Xaa
	1GM9	*Escherichia coli*	penicillin
	1HL7	*Microbacterium sp.*	γ-lactam
	^*^1M21	*Stenotrophomonas maltophilia*	C terminal amide
	^*^1MPL	*Streptomyces sp.*	L-Lys-D-Ala-|-D-Ala
	1MU0	*Thermoplasma acidophilum*	NH-Pro-|-Xaa
	1QTR	*Serratia marcescens*	NH-Pro-|-Xaa
	^*^3A2P	*Arthrobacter sp.*	6-amino exanoate dimer
	3K3W	*Alcaligens faecalis*	penicillin
	^*^3K84	*Rattus norvegicus*	fatty acid amide
	3NWO	*Mycobacterium smegmatis*	NH-Pro-|-Xaa

PDB code of the crystal structures, the source and the natural substrate. References related to the crystal structures are available in Table S1. ^*^Structures that were geometrically reflected before superimposition.

### Calculation of alignment independent BioGPS descriptors

The BioGPS (Global Positioning System in Biological Space) procedure is based on the software FLAP [5] for calculating GRID based molecular descriptors. FLAP uses a “Common Reference Framework” for ligands and proteins, enabling ligand-based and structure-based virtual screening, docking, and 3D-QSAR analysis. BioGPS uses the same approach for comparing protein binding sites. The BioGPS procedure is composed by two main steps: the characterization of the protein active sites and then the comparison by superposing them.

The 42 serine hydrolases listed in Table 1 were used for the calculation of the BioGPS descriptors. Crystal structures of all the dataset enzymes were retrieved from the Protein Data Bank (PDB) [21] and preprocessed by using the software PyMOL [22]: all molecules but the proteins were deleted (i.e. water molecules, inhibitors, glycosylation residues, etc.). The original protein structure coordinates (from the PDB) were used as inputs, without any previous superimposition. First of all the active site of each enzyme was automatically detected by the FLAPsite algorithm [24]. In order to describe the active sites each active site was mapped with the GRID force field [25] for evaluating the type and the energy of non-bonded interactions and then generating the pseudo-MIFs (Molecular Interaction Fields). Four different probes were employed: H probe takes into account the active site shape; O probe that evaluates H-bond donor properties; N1 probe that evaluates the H-bond acceptor capabilities; the DRY probe accounting for hydrophobic interactions. The magnitude of the interaction of the N1 and O probes includes, implicitly, also information about the charge contribution, since these probes have already a partial positive and negative charge respectively.

With the pseudo-MIF procedure, the mapped properties are considered as electron-density like fields centered on each atom, corresponding to specific probe types (i.e. the interaction energies coming from GRID N1 probe were centered on carbonyl oxygen as H-bond acceptor).

First the algorithm reduces the complexity of the pseudo-MIFs selecting a number of representative points using a weighted energy-based and space-coverage function. Then generates all possible combinations of four points; each combination is termed “quadruplet” (in mathematics, a tuple is a finite group of objects and a quadruplet is written as 4-tuple, see Figure S1). Moreover, the function includes the geometrical information into each quadruplet. All possible quadruplets for each mapped active site were generated and stored into a bio-fingerprint (bitstring) that constitutes the Common Reference Framework. For catching similarities and differences between two or more active sites, the algorithm compares their Common Reference Frameworks using an “all against all” approach where each enzyme active site is compared with itself and with all the other enzyme active sites; the algorithm searches for similar quadruplets and then overlaps the corresponding 3D structures. At the end, the algorithm generates a set of Tanimoto scores [26] (BioGPS descriptors) represented by square matrixes, namely a series of probe scores (one for each original GRID probe) together with a global score. The descriptors are calculated for a given superposition by directly comparing the overlapping volumes of the pseudo-MIFs.

### Unsupervised Pattern Cognition Analysis UPCA

Unsupervised Pattern Cognition Analysis (UPCA) is a well established algorithmic platform used for performing systematic analysis of data sets [4]. In this case, the algorithm implemented for performing UPCA clustering is based on Principal Component Analysis (PCA) which is a statistical method commonly used to reduce the dimensionality of data. The UPCA algorithm converts a set of correlated descriptors into a new set of linearly independent variables (orthogonal transformation) called Principal Components (PCs). Principal Components are simply a linear combination of the original correlated variables. The first Principal Component (PC1) is calculated in order to maximize the variance of the object in the dataset. The following principal components are calculated to maximize the variance in the data that is not explained by the previous PC yet. UPCA can easily detect clusters of different active sites for capturing and quantifying differences between protein classes. The main advantage of this approach is represented by the user-friendly visual inspection and analysis of the data making the analysis of similarities and differences very easy and understandable.

The set of Tanimoto scores [26] obtained from BioGPS procedure (square matrixes) was used as input for the UPCA algorithm. In particular five matrices were generated from BioGPS: one for each GRID probe (H, N1, O, DRY) and one considering the global score. In this way five UPCA clustering plot were obtained. By using the global score matrix an overview of the similarities and dissimilarities is achieved. Analyzing a single probe cluster plot, differences in term of one specific non-bonded interaction are highlighted (i.e. by using DRY probes scores matrix differences and similarities in term of hydrophobic interactions are considered).

### CaLB virtual mutants generation

The generation of the CaLB virtual mutants was performed *in-silico* starting from the CaLB structure with the PDB code 1TCA. Amino acids substitution was performed by the mutagenesis tool of the software PyMOL. Each generated mutant was defined inside the GROMOS 53a6 force field [27] and centered inside a cubic system of 343 nm^3^; each system was solvated with explicit SPC water, charges were equilibrated adding Na^+^ and Cl^-^ ions. Afterwards, each system was minimized using the GROMACS software (version 4) [28] and computing 10000 step of steepest descendent gradient. Thus, each minimized system was subjected to a 500 ns of Molecular Dynamic (MD) simulation performed with the software GROMACS (version 4) using an NPT ensemble at 300 K keeping pressure and temperature constant (Berendsen pressure and thermostat) [29], Particle Mesh Ewald (PME) [30] algorithm was used for computing the electrostatic interactions. The output of each MD simulation was carefully analyzed performing a conformational sampling in order to select the proper conformer for each enzyme structure, the sampling was computed with the g_cluster tool of the software GROMACS. At the end of this procedure, each selected conformer was processed by using the software PyMOL: all molecules but the enzyme were deleted (i.e. water molecules and ions).

## Results

### Engineering amidase activity into a lipase scaffold: the rational

Different studies of the last decade have addressed the problem of why proteases/amidases can hydrolyze amides efficiently whereas esterases can not [13]. Lipases, esterases, proteases and amidases are all members of the serine hydrolases superfamily, which is characterized by a serine responsible for the nucleophilic attack of the acyl groups of substrates [31]. The largest part of serine hydrolases adopts an α/β-hydrolase folding and presents a catalytic triad that comprises, besides the nucleophilic serine, two residues responsible for acid/base catalysis [32]. They generally correspond to His and Asp or Glu, although some Ser hydrolases have only a catalytic dyad (Ser/Lys or Ser/His) [33], [34]. These enzymes are also characterized by a region responsible for the stabilization of the oxyanion in the tetrahedral intermediate of the hydrolytic reaction. This active site organization can be found within all serine hydrolases, notwithstanding the extremely low structural or sequence homology. It is, therefore, surprising that esterases/lipases have undetectable or very low amidase activity, as also previously reported in the case of wild type CaLB [20].

In order to understand the structural and chemical basis of these diverse catalytic properties, a series of 42 Ser hydrolases was analyzed. The ultimate aim of this investigation was to gain insights for driving the engineering of amidase activity into the scaffold of lipase B from *Candida antarctica* (CaLB). This lipase is widely employed in industry because of its thermal stability and robustness, so that the introduction of amidase activity into a CaLB scaffold would combine, synergistically, stability and promiscuous specificity of certain industrial interest.

### Preliminary geometrical comparison of active sites based on superimposition

Ser hydrolases belonging to four different enzyme classes, lipases, esterases, proteases and amidases, were considered. The preliminary analysis and comparison of the 42 Ser hydrolase enzymes was performed by superimposing their structure taking the catalytic residues as a reference point. Enzyme structures were obtained from the Protein Data Bank (PDB) [21] and they are listed in Table 1 along with information on their biological source and natural substrates. Further structural details about the residues forming the catalytic apparatus of each enzyme, as well as the references related to each crystal structure, are reported in Table S1. It must be underlined that there are two possible geometrical organizations of active sites of Serine hydrolases, the two of them being specular. Consequently, for 15 enzymes (marked in Table 1) their specular structures were constructed in order to allow the superimposition.

**Figure 1:**
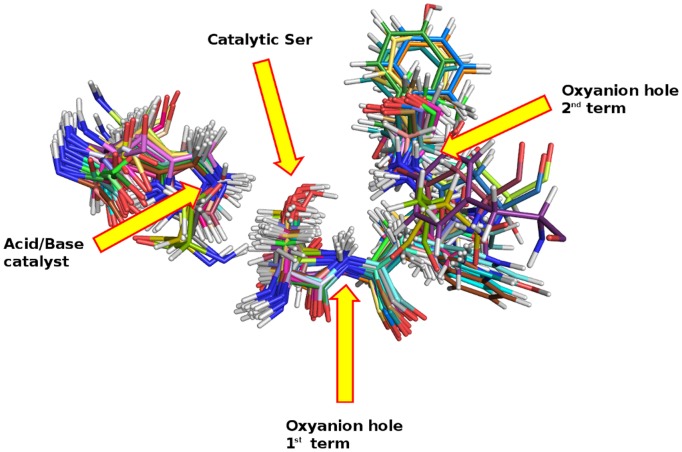
Superimposition of the 42 ser-hydrolases considered and listed in Table 1. All catalytic serines appear superposed at the center of the picture. Arrows highlight the oxyanion hole and the residue responsible for acid-base catalysis.

The 42 structures were superposed taking three structural elements as a reference: the atom acting as the general base during the catalysis (i.e. the Nε2 of His224 in CaLB) and the two H-bond donor residues that constitute the oxyanion hole (i.e. the Nα atoms of Thr40 and Gln106 in CaLB), which are largely conserved within the Ser hydrolases superfamily (for further details see Table S1). The outcome is illustrated in Figure 1, where it is evident how all catalytic serines and the main structural components are well superposed. Overall, the spatial arrangement of the residues responsible for catalysis looks remarkably similar in all 42 enzymes. Conversely, we concluded that structural superimposition seems an inefficient route for revealing the structural properties responsible for the altered mechanism of action or different catalytic efficiency.

In order to explore novel investigation routes, we focused our attention on theories that put emphasis on how the active site of enzymes are tailored for stabilizing transition states mainly via electrostatic interactions [35], [36].

An analysis of enzyme active sites able to account for these properties requires, however, appropriate molecular descriptors, able to account also for physical-chemical properties of the active sites, namely on the hydrophobic/hydrophilic balance and water affinity, since they affect the nature and intensity of electrostatic interactions. For this reason, the GRID based [25] BioGPS molecular descriptors, calculated using the FLAP algorithm [5], were used because they account for geometric and electronic as well as for physical-chemical features of (macro)-molecules, so that all factors are considered as a whole.

### BioGPS molecular descriptors

**Figure 2:**
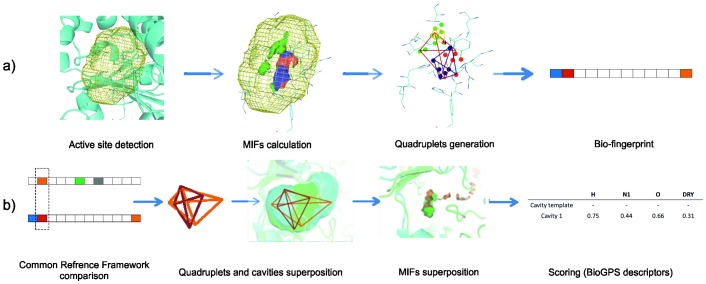
Schematic illustration of the generation of BioGPS molecular descriptors. (a) Starting from the GRID mapping of enzyme active site the BioGPS algorithm identifies points used for generating quadruplets and a Common Reference Framework. (b) In order to compare two cavities (active sites), the algorithm searches for similar quadruplets and then overlaps the corresponding 3D structures (all against all approach). At the end a series of probe scores is generated.

The BioGPS procedure (Global Positioning System in Biological Space) analyzes the protein cavities by means of the GRID based pseudo-molecular interaction fields approach (pseudo-MIF) [37] (Figure 2a), where interaction energies are computed between a chemical probe and the amino acids of the active site. Before the computation of the BioGPS descriptors, the active site of each enzyme was automatically defined by means of the FLAPsite algorithm [24] (Figure 2a) that avoids any manual and arbitrary definition of the active sites while accelerating the operations. It must be underlined that the original protein structure coordinates were used as inputs, without any previous superimposition.

Four chemical probes were chosen for the GRID-mapping and more specifically: the H probe, taking into account the active site shape; O probe that evaluates mainly H-bond donor properties; N1 probe that estimates mainly the H-bond acceptor capabilities; the DRY probe, accounting for hydrophobic interactions. The output of the GRID-mapping (Molecular Interaction Fields or MIFs) corresponds to a “negative” picture of interactions that are likely to occur inside the active site, thus contributing to the stabilization of the transition state of a given reaction. The procedure computes energetically favorable but also unfavorable interactions and indicates the direction for engineering a desired catalytic activity inside an enzyme scaffold by mimicking an environment where specific residues supply the necessary interactions.

The BioGPS algorithm condenses the information contained in the GRID-pseudoMIF_s_ into a common reference framework of four-point fingerprints called “quadruplets” (see Figure S1). From the mathematic point of view, a “tuple” corresponds to a finite group of objects, so that a quadruplet is written as 4-tuple. The algorithm uses a weighted energy function that allows overcoming the differences in absolute value between electrostatic and hydrophobic interactions. The BioGPS algorithm generates all possible quadruplets inside the active sites and the function includes the geometrical information into each quadruplet.

Information, contained in the quadruplets and mathematically associated to bitstrings, or better bio-fingerprints (Figure S1), were finally compared within a Common Reference Framework with the ultimate aim of disclosing similarities and differences between two or more cavities (active sites). More in detail, the algorithm searches for similar quadruplets with an “all against all” approach and then the corresponding 3D structure are overlapped aligning their corresponding quadruplets. The all against all approach compares each enzyme active site with itself and with all the other protein active sites. The output is represented by different square matrixes which represent the BioGPS descriptors, namely a series of probe scores (one for each original GRID probe) together with a global score (Figure 2b).

The information contained in the BioGPS descriptors was then statistically analyzed by means of Unsupervised Pattern Cognition Analysis (UPCA) [4]. Ser hydrolases were sorted and grouped into clusters and it is evident that for each class of Ser hydrolases the structural properties explained by the BioGPS descriptors are correlated with catalytic functions (e.g. amidase catalytic activity). Moreover, the multivariate statistical analysis allows also for the “unfolding” of the information contained in the BioGPS descriptors, thus providing rational and quantitative guidelines for engineering promiscuous amidase activity into lipase scaffolds, as described in the following paragraphs.

## Discussion

### Unsupervised Pattern Cognition Analysis (UPCA)

**Figure 3:**
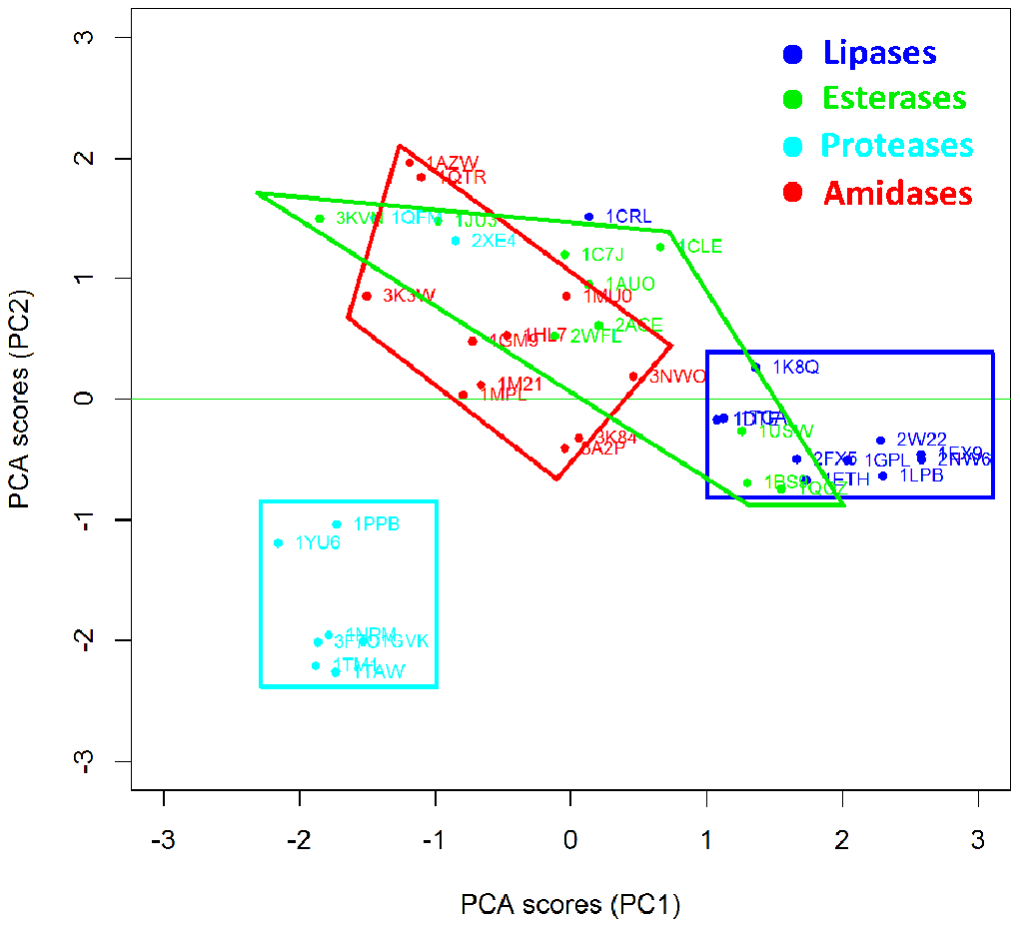
Unsupervised Pattern Cognition Analysis (UPCA) and clustering of Ser hydrolases on the basis of BioGPS descriptors (global score). The enzymes are labelled according to their PDB code. Lipases are indicated in blue, esterases in green, amidases in red and proteases in cyan.

Clustering methods are based on the application of machine learning techniques to identify inherent patterns in a data set [4]. More specifically, in the present work Unsupervised Pattern Cognition Analysis (UPCA) was applied to perform unbiased grouping of the enzymes on the basis of the similarity matrixes coming from the BioGPS descriptors (Figure 3). The global score of the Pattern Cognition Analysis groups the objects on the firsts two Principal Components (PCs), namely the latent variables, which explain 23% of the whole variance (PC1 = 14%; PC2 = 9%). PC3 explains only 4% of the variance, so that two PC appears sufficient to explain the differences among active sites, whereas the remaining 77% of variance has to be considered as noise or diversity that is not explained by this model. It is important to underline that UPCA is not a regression analysis, therefore it just analyzes the already existent correlation of variables without searching for the maximum correlation. Therefore, the remaining 77% of variance is most probably ascribable to heterogeneous substrate specificity, which appears as a predominant cause of variability inside Ser-hydrolase superfamily.

Figure 3 demonstrates that the BioGPS-UPCA procedure groups proteases, lipases and amidases into clearly distinct clusters whereas esterases appear as a rather heterogeneous class of hydrolases. It is important to note that the enzymes used in the data set (Table 1) are listed and classified according to their annotated E.C. number. Although this classification is sometimes object of debate among enzymologist community because of the lack of quality control, it is consistent with the objective of the present investigation. In fact, all enzymes are classified on the basis of their main catalytic activities, although some of them are well known for their promiscuity. As an example, 1GM9 (penicillin G amidase) is also employed in biocatalysis for its ability to catalyze the aminolysis and hydrolysis - although not the synthesis – of phenylacetic acid esters [38], [39] That experimental evidence is reflected by the localization of 1GM9 within the amidase area but at the interface with the esterase group.

Notably, proteases are grouped in a region clearly distinct from amidases, although the two classes are very much related. Therefore, BioGPS descriptors are able to explain the differences, as well as shared features, of the four hydrolases classes, in spite of the heterogeneous substrate specificity represented within each class.

Interestingly, 1CRL (lipase from *Candida rugosa*) is located near 1CLE (Cholesterol esterase from *Candida cylindracea*). These two enzymes are different in terms of reaction and substrate selectivity but they have a sequence homology higher than 40% [40] (enzyme clustering based on RMSD sequence backbone structure similarity is available in Figure S2).

Two proteases (1QMF and 2XE4) result as outliers and their behavior has been analyzed in detail by studying the scores coming from the O probe (see section below O probe (H-bond donor properties)).

### Test and validation of BioGPS-UPCA: global score

The reliability of the BioGPS-UPCA approach and its potential application to *in silico* enzyme design and screening was verified by “projecting” CaLB mutants into the UPCA domain. The structures of eight CaLB mutants, engineered with the aim of introducing promiscuous amidase activity, were taken from the literature [20], [41] and processed for the extraction of the BioGPS descriptors. The UPCA analysis located the mutants according to their new structural properties, which are significantly correlated to their experimentally determined amidase activities (hydrolysis of N-benzyl-2-chloroacetamide) expressed as improvement factor referred to CaLB wild type [20] (Table 2). It must be underlined that the wild type CaLB has a poor amidase activity, especially on the considered substrate (specific activity of CaLB wild type = 1.27±0.16×10^−2^ µmol/mg/h) and the improvement factor is defined as the ratio between the specific activity of each mutant and the specific activity of the wild type CaLB [20].

**Table 2:** CaLB mutants used for the validation of the BioGPS-UPCA model and taken from ref 20 and ref 39.

Mutant	Mutation	Improvement factor - IF (Relative amidase activity)
M1	G39A/W104F/L278A	6.3
M2	G39A/T103G/L278A	3.8
M3	G39A/T103G/W104F/L278A	11.2
M4	G39A	2.8
M5	G39A/L278A	3.3
M6	I189A	0.4
M7	T40A	0.4
M8	T103G	1.1

The amidase activities are expressed as improvement factor (IF) referred to CaLB wild type activity: IF = Amidase activity of mutant/Amidase activity of CaLB wild.

The models of these mutants were *in-silico* generated using as a template the structure of the wild type CaLB 1TCA. The mutations were introduced by the mutagenesis tool of the software PyMOL [22], mutants structures were defined into GROMOS 53a6 force field [27], minimized with a steepest descendent algorithm and relaxed by 500 ns of Molecular Dynamic (MD) simulation performed with the software GROMACS [28]. Each MD trajectory was carefully evaluated by performing a conformational sampling analysis. Particular attention was given to this procedure in order to select the most representative mutant conformer.

**Figure 4:**
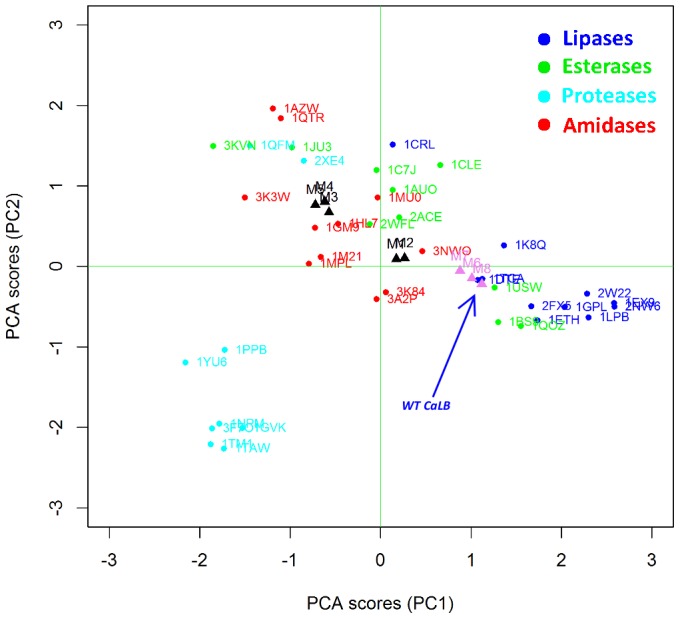
Projection of CaLB mutants in the BioGPS-UPCA model (global score). Improved mutants are highlighted as black triangles whereas poor mutants are in pink. WT CaLB (1TCA) is indicated by the blue arrow. The different classes of Ser hydrolases are reported in different colors, as also described in Figure 3.

Interestingly, CaLB mutants that have an improved amidase activity (M1-5, black triangles in Figure 4) shift significantly towards the amidase cluster whereas poor mutants (M6-8, pink triangles in Figure 4) remain close to the WT position inside the lipase cluster. Results indicate that BioGPS-UPCA procedure is effective in extracting relevant information from the 3D enzyme structures and, more importantly, such information is correlated to the ability of the active site to stabilize the transition state for the hydrolysis of the amide bond, This first application of BioGPS-PCA to bioinformatic analysis opens new perspectives towards the *in-silico* screening of virtual mutants potentially endowed with activities of interest.

A better understanding of the factors that determine the localization of a given enzyme structure inside a defined cluster can be achieved by analyzing the BioGPS descriptors resulting from each different probe score (one for each original GRID probe). This detailed analysis provides guidelines for driving rational strategies for mutagenesis.

### H probe (active site shape)

**Figure 5:**
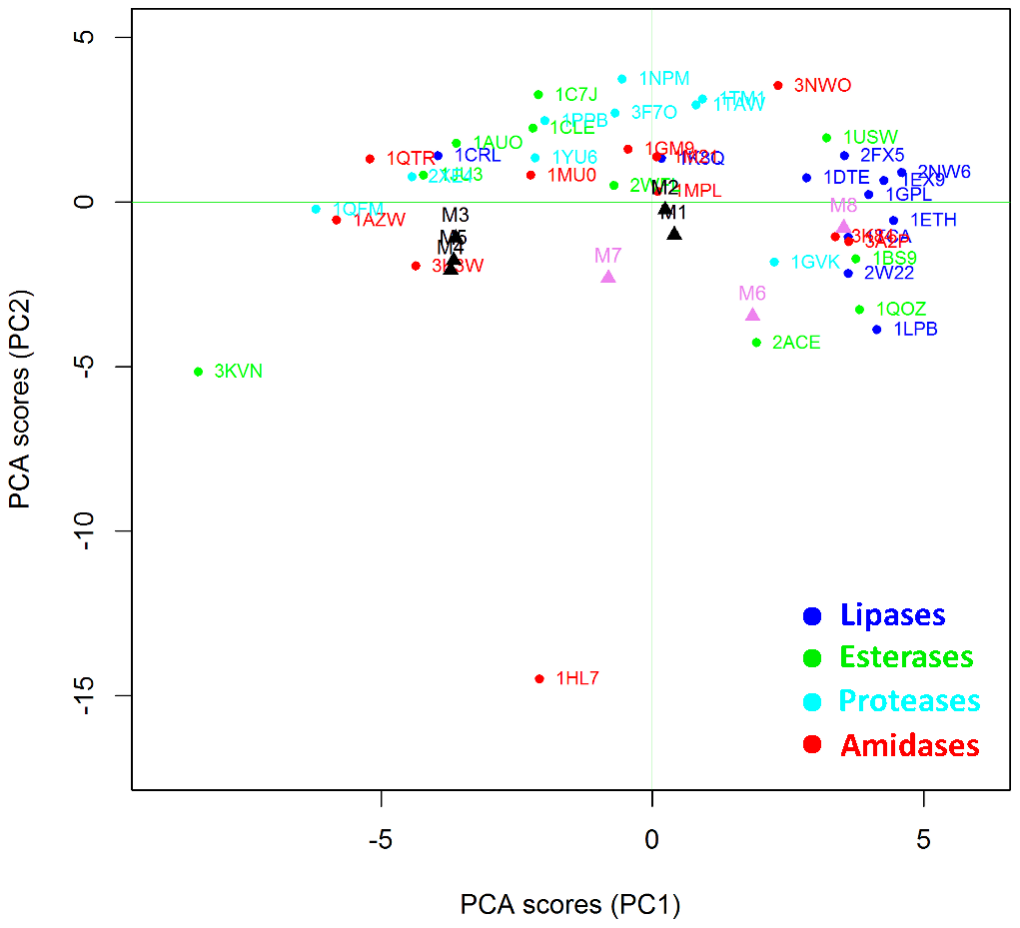
Unsupervised Pattern Cognition Analysis (UPCA) of BioGPS descriptors generated by H probe (shape). The enzymes are labelled according to their PDB code and colored as in figure 3. Improved mutants are highlighted in black triangles and poor mutants are in pink triangles.

The active sites of all 42 enzymes were mapped by means of the H probe, which mainly describes the volume and the shape of the chemical target. The bio-fingerprints, calculated by the BioGPS algorithm, were used for the alignment and comparison of the active sites. Figure 5 shows clearly that the shape probe alone is not able to classify the different Ser hydrolases. All Ser hydrolases appears overlapped, although lipases are grouped on the right side of PCA domain. The two outliers in Figure 5 correspond to an amidases with a narrow active site access (1HL7, amidase from *Micobacterium sp.*) and an esterase with a very superficial active site (3KVN, esterase from *Pseudomonas aeruginosa*).

**Figure 6:**
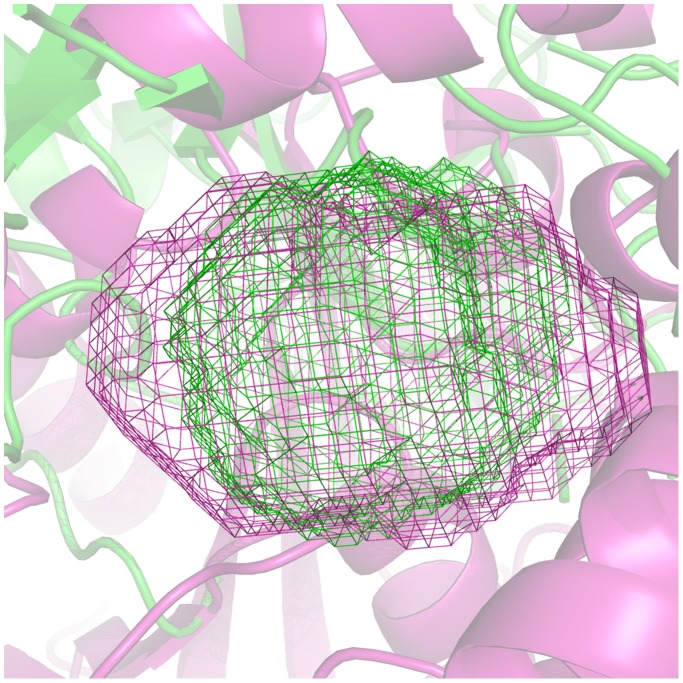
Comparison of 1GVK (protease) and 2W22 (lipase) active site shape. 1GVK and 2W22 are represented as green and magenta cartoon respectively. Active site shapes are represented as wireframes: 1GVK active site shape in green while the active site shape of 2W22 is in magenta.

Figure 6 illustrates the wireframes corresponding to the active site volumes of protease from *Sus scrofa* (1GV6) and lipase from *Geobacillus thermocatenulatus* (2W22). The example indicates a significant similarity, despite the fact the two hydrolases belong to different classes.

### O probe (H-bond donor properties)

**Figure 7:**
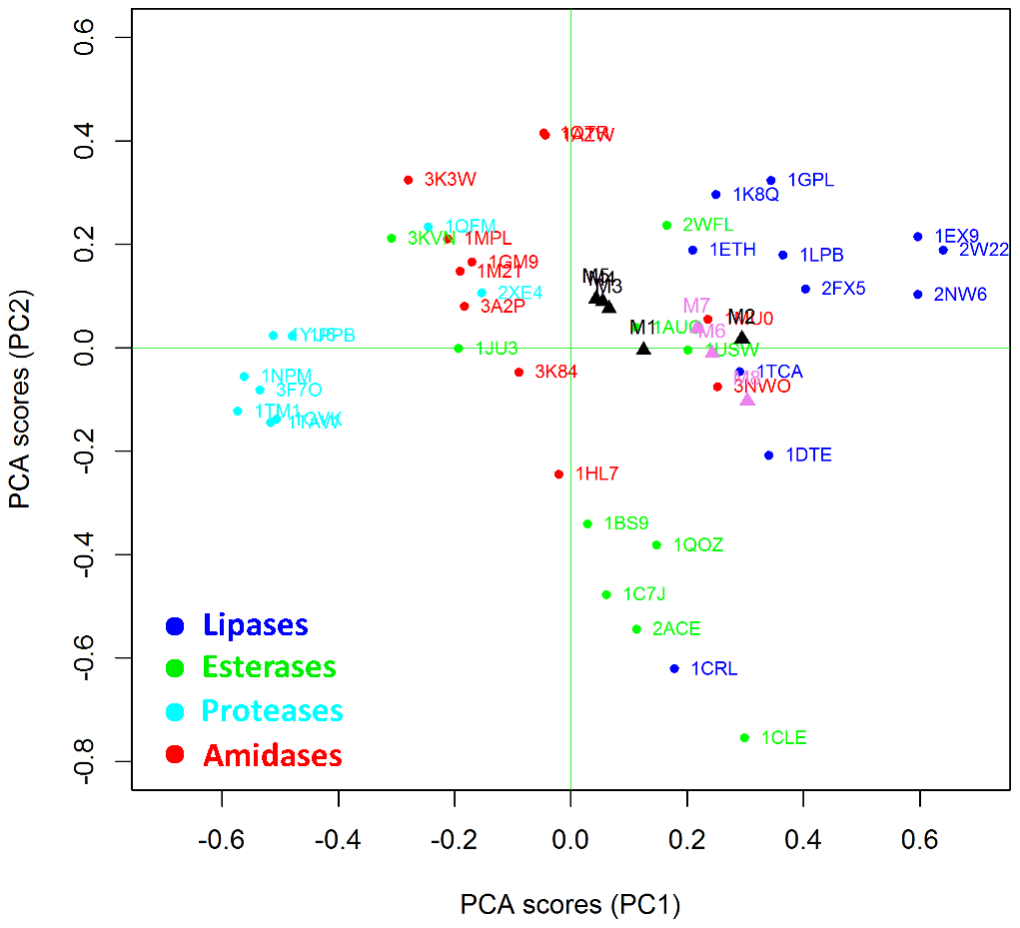
Unsupervised Pattern Cognition Analysis (UPCA) of BioGPS descriptors generated by O probe (H-bond donor capacity). The analyzed enzymes are labelled according to their PDB code and colored as in figure 3. Improved mutants are highlighted in black triangles whereas poor mutants are in pink triangles.

The UPCA analysis of the BioGPS descriptors generated by the O probe identifies the four Ser hydrolases classes (Figure 7), although amidases (red) and esterases (green) appears mostly overlapped. The fact that peptidases and lipases are clearly separated along the first PC suggests that the ability of the enzymes to donate H-bonds increases moving to the left. The interpretation of the second PC is more complex and it might be related to the geometrical distribution of the H-bond donor species inside the active sites.

Proteases appear, once again, as the most distinct and discriminated class, although there are two outliers falling into the amidase cluster (1QMF and 2XE4). Consequently, their structural differences are not explained by the PC because they present specific features, which are not shared by the protease class. For instance, 1QMF (protease from *Sus scrofa*) is the only protease of the data set able to hydrolyze peptides in correspondence of Pro residues [42]. Enzyme 2XE4 is a protease from *Leshmania major* and it catalyzes the hydrolysis of much shorter olygopeptides as compared to the other proteases of the data set. Moreover, these two proteases present a Tyr residue in the oxyanion hole (Y473 and Y496 for 1QMF and 2XE4 respectively), where the oxygen of the OH group of Tyr stabilizes the tetrahedral intermediate acting as a H-bond acceptor, thus replacing the function of the amide nitrogen present in all the other structures.

Interestingly, lipase from *Humicola lanuginosa* (1DTE), lipase from *Candida rugosa* (1CRL) and lipase from *Candida Antarctica* (CaLB, 1TCA), are localized closer to the amidase cluster as compared to other lipases. Therefore, according to the H-bond donor properties, CaLB appears as suitable scaffolds for introducing promiscuous amidase activity. Indeed, CaLB it has been always considered as a non conventional lipase since it does not display interfacial activation and accepts short chain fatty acids and Figure 7 highlights how it is structurally recognized as member of the esterase cluster [43].

Figure 7 shows how the CaLB mutants endowed with higher amidase activity (M1–M5) fall within the amidase region, indicating that mutations were effective in modifying the H-bond donor capacity in the direction of improving amidase activity. Poorly active mutants remain close to the WT position, suggesting no significant variations in terms of H-bond donor capacity.

It must be underlined that the effect of the mutations cannot be interpreted simply by comparing the structural properties of each single residue, since the phenotype is the result of a complex array of physical-chemical, geometrical and electronic variations and interactions. As an example, in M4 Gly39 is replaced by an Ala, a residue that, in principle, does not provide extra H-bond donor groups. However, such mutation can rather modify the geometrical organization of the active site and its ability to establish H-bonds.

**Figure 8:**
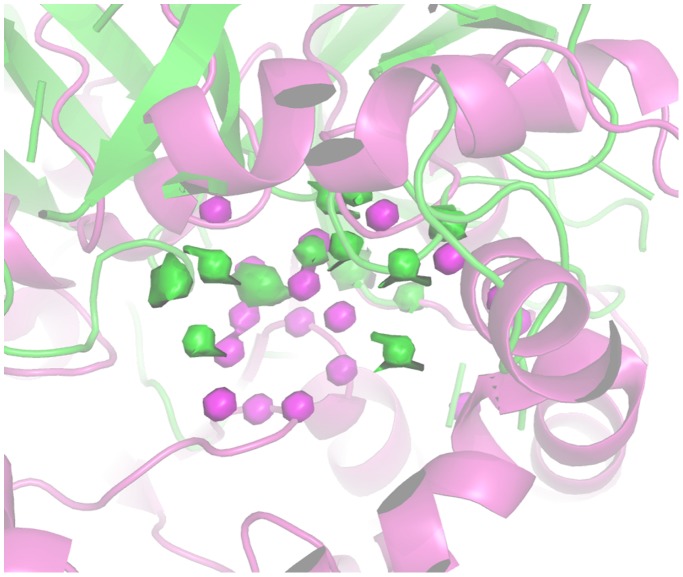
Comparison of 1GVK (protease) and 2W22 (lipase) active site H-bond donor pseudo-MIFs. The structures of 1GVK and 2W22 are represented in green and magenta cartoons respectively. 1GVK pseudo-MIFs are represented as green surfaces. 2W22 pseudo-MIFs are represented as magenta surfaces.

Figure 8 reports the pseudo-MIFs of a protease (1GVK) and a lipase (2W22). The superimposition was driven by the alignment of the corresponding quadruplets, so that the extension and position of the pseudo-MIFs provide a visual description of differences between the two enzymes in terms of ability to donate H bonds.

### N1 probe (H-bond acceptor properties)

**Figure 9:**
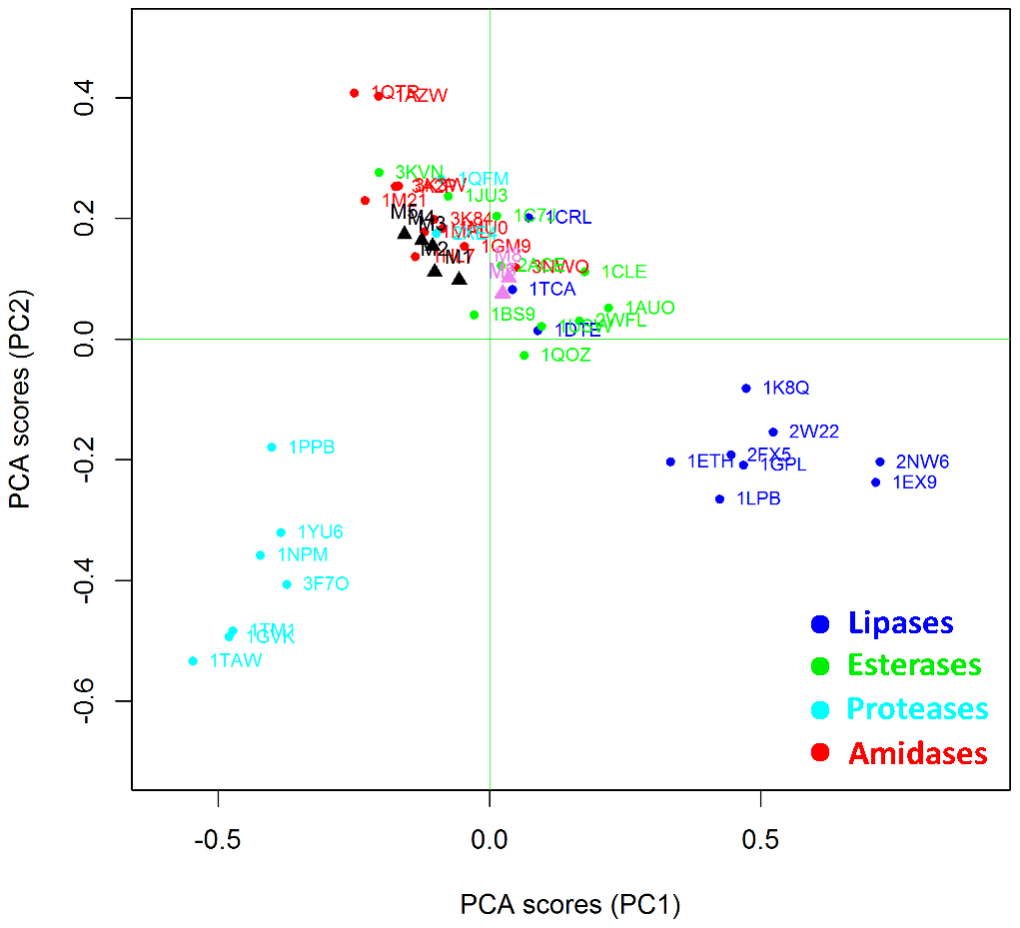
Unsupervised Pattern Cognition Analysis (UPCA) of BioGPS descriptors generated by N1 probe (H-bond acceptor capabilities). The analyzed enzymes are labelled according to their PDB code and colored as in figure 3. Improved mutants are highlighted as black triangles and poor mutants are pink triangles.

The UPCA analysis of the BioGPS descriptors generated by the N1 probe is observable in Figure 9.

As in the previous case, proteases and lipases are completely separated on the basis of their ability to establish H-bonds (first PC). It is noteworthy that amidases and esterases have distinct H bond acceptor properties, whereas in the case of O probe the two classes resulted overlapped. This indicates that engineering amidase activity into esterases requires the improving of H bond acceptor ability of the active sites. Lipase from *Candida rugosa* (1CRL) behaves, again, as an outlier: it clearly localized within esterase area but it falls far from the amidase cluster.

**Figure 10:**
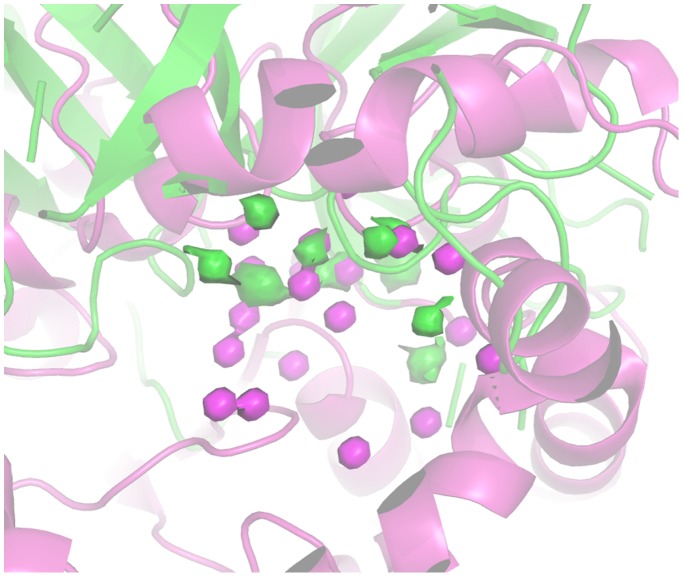
Comparison of 1GVK (protease) and 2W22 (lipase) active site H-bond acceptor pseudo-MIFs. 1GVK and 2W22 are represented as green and magenta cartoon respectively. 1GVK pseudo-MIFs are represented as green surfaces. 2W22 pseudo-MIFs are represented as magenta surfaces.

Mutants M1, M3, M4 and M5 move significantly towards esterases and amidases whereas the low active mutants M7 and M8 remain close to the WT. It must be noted that also M2 remains close to WT, although it is a good mutant (IF = 3.8) and this indicates how the combination of different factors, accounted by different probes, determines the global improvement of the amidase activity. Figure 10 reports the comparison between protease 1GVK and lipase 2W22 in terms of their ability to accept H-bonds.

### DRY probe (hydrophobicity)

**Figure 11:**
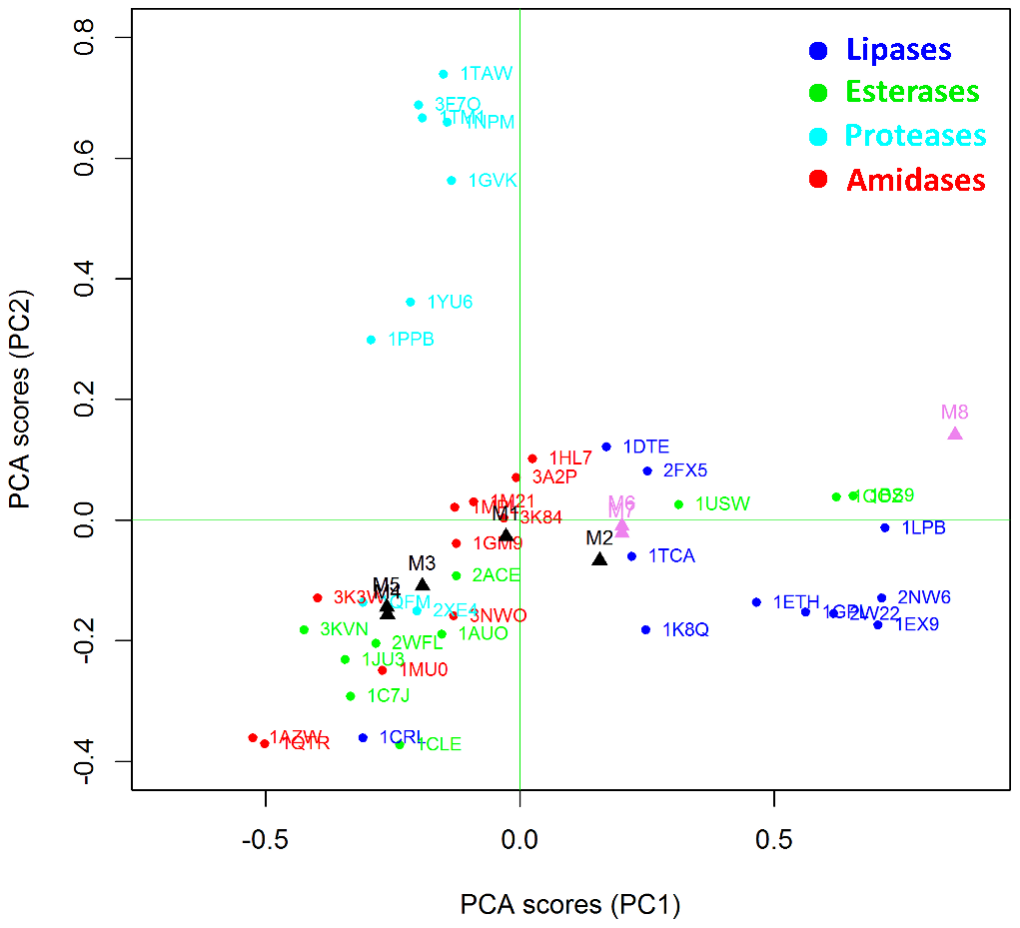
Unsupervised Pattern Cognition Analysis (UPCA) of BioGPS descriptors generated by DRY probe (hydrophobicity). The analyzed enzymes are labelled according to their PDB code and colored as in figure 3. Improved mutants are highlighted in black triangles and poor mutants are in pink triangles.

The UPCA analysis of the BioGPS descriptors generated by the DRY (Figure 11) highlight, as expected, the distinct hydrophobic nature of the active sites of lipases, as they accept fatty acids and triglycerides. Interestingly, the active site of lipase from *Candida rugosa* (1CRL) appears far less hydrophobic, and this observation confirms that CRL is endowed with uncommon structural features as compared to the clustered lipases.

**Figure 12:**
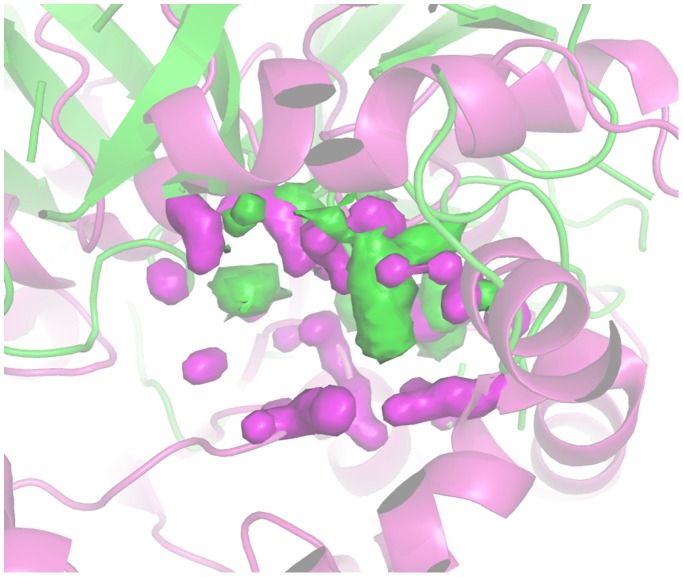
Comparison of 1GVK (protease) and 2W22 (lipase) active site hydrophobic pseudo-MIFs. 1GVK and 2W22 are represented as green and magenta cartoon respectively. 1GVK pseudo-MIFs are represented as green surfaces. 2W22 pseudo-MIFs are represented in magenta.

The comparison of the active sites of protease 1GVK and lipase 2W22 reported in Figure 12 makes evident the extended hydrophobicity of the lipase active site.

The projection of mutants in the UPCA domain (Figure 11) indicates that the positive mutations introduced in the improved mutants M1, M3, M4 and M5 clearly induce a decrease of hydrophobicity in the active sites. Once again, this variation of properties cannot be ascribed to a single structural element or residue, but rather it comes from the complex combination of different factors that cannot be analyzed singularly.

## Conclusions

A computational methodology was developed based on the Unsupervised Pattern Cognition Analysis (UPCA) of GRID-based BioGPS descriptors (Global Positioning System in Biological Space) that allows for the clustering of enzymes and mutants through the 3D-structural bioinformatic analysis and comparison of their active sites. As compared to classical bioinformatic analysis, the BioGPS-UPCA method does not rely on simple sequence alignment or pre-alignment of protein structures.

The method was validated by considering its ability to predict the effect of mutation in CaLB variants produced with the aim of engineering amidase activity into a lipase scaffold. The efficiency of this new structure-based bioinformatic strategy was demonstrated by the consistent grouping of four different Ser hydrolase classes inside distinct clusters and areas. These results indicate that, notwithstanding all enzymes considered are characterized by the same active site organization, the BioGPS-UPCA method recognized the structural elements that make hydrolases able to catalyze different reactions. More importantly, the BioGPS-UPCA model predicted correctly the properties of lipase mutants endowed with improved amidase activity. The projection of mutants endowed with novel catalytic properties into the UPCA domain demonstrates that the new structural features introduced into the lipase scaffold are correlated with the catalytic properties of the enzymes. The results support the predominant role of electrostatic interactions in the stabilization of the transition states of reactions here considered [36].

The BioGPS-UPCA methodology allows also for the “unfolding” of the information contained in the BioGPS descriptors. Structural, physical-chemical and electrostatic factors, which are shared by a specific enzyme class, were analyzed in detail, thus providing guidelines for rational engineering strategies. Furthermore, the clustering of the different Ser hydrolases classes (Figure 3) is based on an ensemble of different physical-chemical and electrostatic properties and a comprehensive analysis can be performed only by considering all these factors and their interactions at the same time. Indeed, when each property is considered at a time, the clustering is not so evident, because only a partial analysis is provided. Nevertheless, the information coming from each single probe can be exploited for guiding the insertion of a specific property inside an active site by protein engineering.

In conclusion, BioGPS descriptors are effective in accounting for geometric, electronic and physical-chemical factors and open new perspectives for 3D-phylogenetic analysis and unbiased *in-silico* screening of virtual mutants. The method groups enzymes on the bases of similarities and differences but also provides focused insights for guiding the rational re-design of specific physical-chemical and electrostatic properties into their active sites. Therefore, BioGPS-UPCA approach represents an actual tool for translating the massive amount of databases information into valuable and usable knowledge.

## Supporting Information

Figure S1 Quadruplets definition according to BioGPS algorithm; each quadruplet is defined as a bitstring as indicated at the bottom of the picture.
(TIF)

Figure S2 Dataset clustering based on RMSD multidimensional scaling. Each structure is projected according to RMSD structure similarity (referred to the same structure and to all the other enzymes). The RMSD was calculated by superposing the backbone atoms of each enzyme structure.
(TIF)

Table S1 Ser hydrolases analyzed, for each crystal structure the residues used for the catalytic machinery based superimposition are indicated.
(DOC)
