# Supplementary figures and images for: BioGPS Descriptors for Rational Engineering of Enzyme Promiscuity and Structure Based Bioinformatic Analysis
Source: PLoS One. 2014 Oct 29;9(10):e109354. doi: 10.1371/journal.pone.0109354 (PMC4212942; doi:10.1371/journal.pone.0109354)

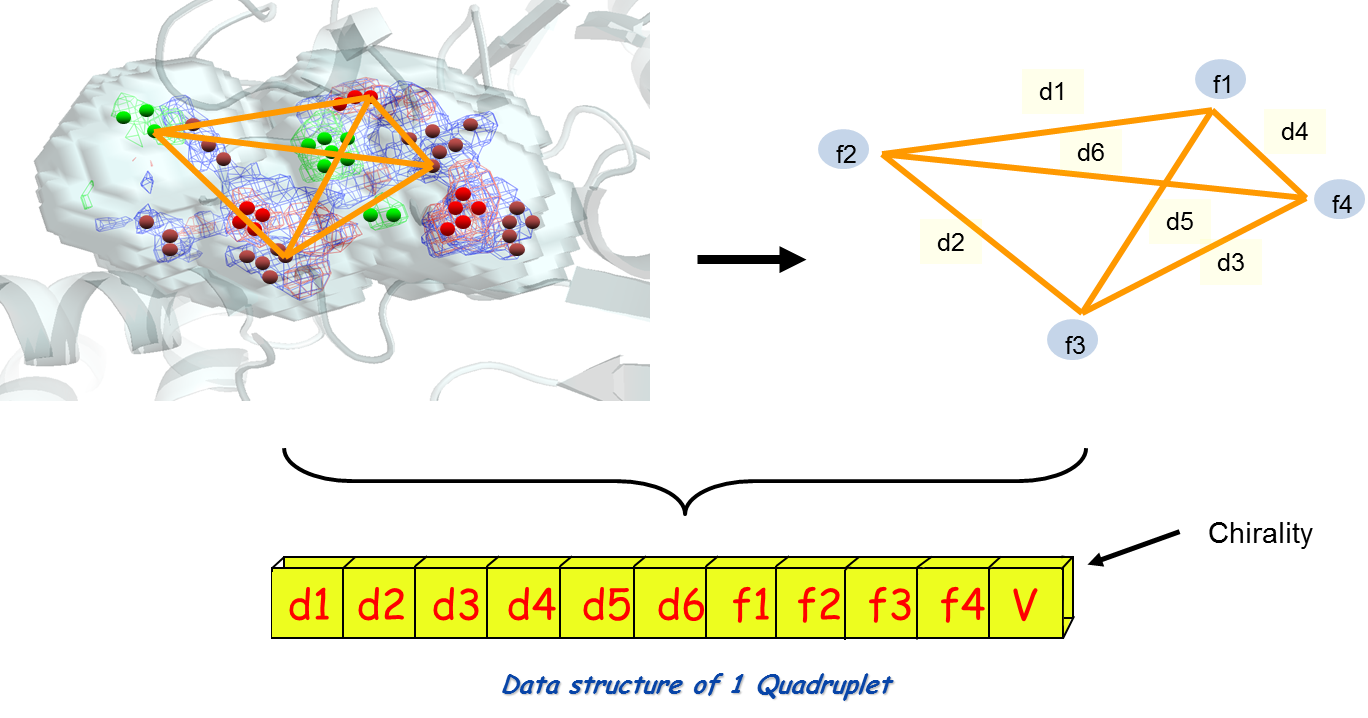

Supplement: Figure S1 — Quadruplets definition according to BioGPS algorithm; each quadruplet is defined as a bitstring as indicated at the bottom of the picture. (TIF) [file pone.0109354.s001.tif]

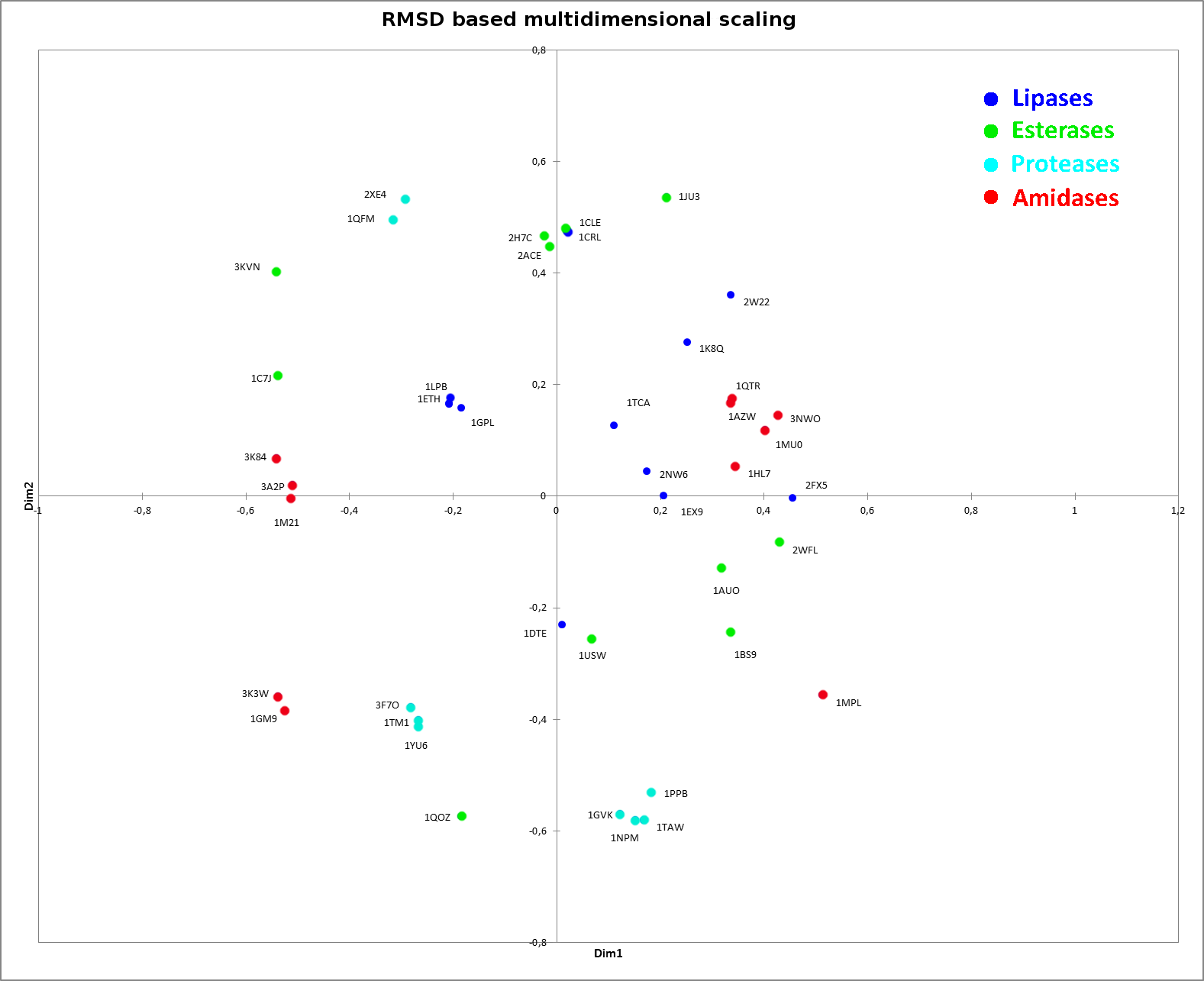

Supplement: Figure S2 — Dataset clustering based on RMSD multidimensional scaling. Each structure is projected according to RMSD structure similarity (referred to the same structure and to all the other enzymes). The RMSD was calculated by superposing the backbone atoms of each enzyme structure. (TIF) [file pone.0109354.s002.tif]
