# Supplementary material for: BioGPS Descriptors for Rational Engineering of Enzyme Promiscuity and Structure Based Bioinformatic Analysis
Source: PLoS One. 2014 Oct 29;9(10):e109354. doi: 10.1371/journal.pone.0109354 (PMC4212942; doi:10.1371/journal.pone.0109354)
Supplement: Table S1 — Ser hydrolases analyzed, for each crystal structure the residues used for the catalytic machinery based superimposition are indicated. (DOC) [file pone.0109354.s003.doc]

**Table S1.** Ser hydrolases analyzed, for each crystal structure the residues used for the catalytic machinery based superimposition are indicated.

| **Enzyme class** | **PDB code** | **Acid/base**  **catalyst** | **Oxianion**  **1^st^ term** | **Oxianion**  **2^nd^ term** | **Catalytic**  **Ser** |
| --- | --- | --- | --- | --- | --- |
| Lipases | 1CRL[[1]](#endnote-2) | His449 | Ala210 | Gly124 | 209 |
|  | 1DTE[[2]](#endnote-3) | His258 | Leu147 | Ser83 | 146 |
|  | 1ETH[[3]](#endnote-4) | His263 A | Leu153 A | Phe77 A | 152 A |
|  | 1EX9[[4]](#endnote-5) | His251 | His83 | Met16 | 82 |
|  | 1GPL[[5]](#endnote-6) | His263 | Leu153 | Phe77 | 152 |
|  | 1K8Q[[6]](#endnote-7) | His353 | Gln154 | Leu67 | 153 |
|  | 1LPB[[7]](#endnote-8) | His363 | Leu153 | Phe77 | 152 |
|  | 1TCA[[8]](#endnote-9) | His224 | Gln106 | Thr40 | 105 |
|  | 2FX5[[9]](#endnote-10) | His206 | Gln127 | Thr58 | 126 |
|  | 2NW6[[10]](#endnote-11) | His286 | Gln88 | Leu17 | 87 |
|  | 2W22[[11]](#endnote-12) | His359 | Gln115 | Phe17 | 114 |
| Esterases | 1AUO[[12]](#endnote-13) | His199 | Gln115 | Leu23 | 114 |
|  | 1BS9[[13]](#endnote-14) | His187 | Gln91 | Thr13 | 90 |
|  | 1C7J[[14]](#endnote-15) | His399 | Ala190 | Ala107 | 189 |
|  | 1CLE[[15]](#endnote-16) | His449 | Ala210 | Gly123 | 209 |
|  | 1JU3[[16]](#endnote-17) | His287 | Tyr118 | Tyr44 | 117 |
|  | 1QOZ[[17]](#endnote-18) | His187 | Gln91 | Thr13 | 90 |
|  | 1USW[[18]](#endnote-19) | His247 | Leu134 | Thr68 | 133 |
|  | 2ACE[[19]](#endnote-20) | His440 | Ala201 | Gly118 | 200 |
|  | 2H7C[[20]](#endnote-21) | His1468 | Ala1222 | Gly1143 | 1221 |
|  | 2WFL[[21]](#endnote-22) | His289 | Phe88 | Gly19 | 87 |
|  | 3KVN[[22]](#endnote-23) | His57 | Ser14 | Gly25 | 14 |
| Proteases | 1GVK[[23]](#endnote-24) | His57 | Ser195 | Gly193 | 195 |
|  | 1NPM[[24]](#endnote-25) | His57 | Ser195 | Gly193 | 195 |
|  | 1PPB[[25]](#endnote-26) | His57 | Ser195 | Gly193 | 195 |
|  | 1QFM[[26]](#endnote-27) | His680 | Asn555 | Tyr473 | 554 |
|  | 1TAW[[27]](#endnote-28) | His57 | Ser195 | Gly193 | 195 |
|  | 1TM1[[28]](#endnote-29) | His64 | Ser221 | Asn155 | 221 |
|  | 1YU6[[29]](#endnote-30) | His64 | Ser221 | Asn155 | 221 |
|  | 2XE4[[30]](#endnote-31) | His697 | Ala578 | Tyr496 | 577 |
|  | 3F7O[[31]](#endnote-32) | His72 | Ser227 | Asn164 | 227 |
| Amidases | 1AZW[[32]](#endnote-33) | His294 | Trp111 | Gly43 | 110 |
|  | 1GM9[[33]](#endnote-34) |  | Ala69 B | Asn241 B | 1 B |
|  | 1HL7[[34]](#endnote-35) | His259 | Met99 | Tyr32 | 98 |
|  | 1M21[[35]](#endnote-36) | Ser202 | Ser226 | Asp224 | 226 |
|  | 1MPL[[36]](#endnote-37) |  | Ser62 | Tyr301 | 62 |
|  | 1MU0[[37]](#endnote-38) | His271 | Tyr106 | Gly37 | 105 |
|  | 1QTR[[38]](#endnote-39) | His296 | Trp114 | Gly46 | 113 |
|  | 3A2P[[39]](#endnote-40) | Ser150 | Ser174 | Ala172 | 174 |
|  | 3K3W[[40]](#endnote-41) |  | Ala69 B | Asn241 B | 1 B |
|  | 3K84[[41]](#endnote-42) | Ser217 | Ser241 | Ile238 | 241 |
|  | 3NWO[[42]](#endnote-43) | His280 | Trp114 | Gly42 | 113 |

1. Grochulski P, et al. (1993) Insights into interfacial activation from an open structure of *Candida rugosa* lipase. *J Biol Chem* 268(17): 12843-12847. [↑](#endnote-ref-2)
2. Brzozowski, A. M. *et al.* Structural origins of the interfacial activation in *Thermomyces* (*Humicola*) *lanuginosa* lipase. *Biochemistry* **39**, 15071-15082 (2000). [↑](#endnote-ref-3)
3. Hermoso, J. *et al.* Lipase activation by nonionic detergents. The crystal structure of the porcine lipase-colipase-tetraethylene glycol monooctyl ether complex. *J. Biol. Chem.* **271**, 18007-18016 (1996). [↑](#endnote-ref-4)
4. Nardini M, Lang DA, Liebeton K, Jeager KE, Dijkstra BW, (2000) Crystal structure of *Pseudomonas aeruginosa* lipase in the open conformation. The prototype for family I.1 of bacterial lipases. *J Biol Chem* 275(40): 31219-31225. [↑](#endnote-ref-5)
5. Withers-Martinez C, Carriere F, Verger R, Bourgeois D, Cambillau C (1996) A pancreatic lipase with a phospholipase A1 activity: crystal structure of a chimeric pancreatic lipase-related protein 2 from guinea pig. *Structure* 4(11): 1363-1374. [↑](#endnote-ref-6)
6. Roussel A, et al. (2002) Crystal structure of the open form of dog gastric lipase in complex with a phosphonate inhibitor. *J Biol Chem* 277(3): 2266-2274. [↑](#endnote-ref-7)
7. Elgoff MP, et al. (1995) The 2.46 A resolution structure of the pancreatic lipase-colipase complex inhibited by a C11 alkyl phosphonate. *Biochemistry* 34(9): 2751-2762. [↑](#endnote-ref-8)
8. Uppenberg J, Hasen MT, Patkar S, Jones TA (1994) The sequence, crystal structure determination and refinement of two crystal forms of lipase B from *Candida antarctica*. *Structure* 2(4): 293-308. [↑](#endnote-ref-9)
9. **DOI:**10.2210/pdb2fx5/pdb [↑](#endnote-ref-10)
10. Luic M, et al. (2008) Combined X-ray diffraction and QM/MM study of the *Burkholderia cepacia* lipase-catalyzed secondary alcohol esterification. *J Phys Chem B* 112(16): 4876-4883. [↑](#endnote-ref-11)
11. Carrasco-Lopez C, et al. (2009) Activation of bacterial thermoalkalophilic lipases is spurred by dramatic structural rearrangements. *J Biol Chem* 284(7): 4365-4372. [↑](#endnote-ref-12)
12. Kim KK, et al. (1997) Crystal structure of carboxylesterase from *Pseudomonas fluorescens*, an alpha/beta hydrolase with broad substrate specificity. *Structure* 5(12): 1571-1584. [↑](#endnote-ref-13)
13. Ghosh D, et al. (1999) Determination of a protein structure by iodination: the structure of iodinated acetylxylan esterase. A*cta Crystallogr D* 55(Pt 4): 779-784. [↑](#endnote-ref-14)
14. Spiller B, Gershenson A, Arnold FH, Stevens RC (1999) A structural view of evolutionary divergence. *P Natl Acad Sci USA* 96(22): 12305-12310. [↑](#endnote-ref-15)
15. Ghosh D, et al. (1995) Structure of uncomplexed and linoleate-bound *Candida cylindracea* cholesterol esterase. *Structure* 3(3): 279-288. [↑](#endnote-ref-16)
16. Larsen NA, et al. (2002) Crystal structure of a bacterial cocaine esterase. *Nat Struct Biol* 9(1): 17-21. [↑](#endnote-ref-17)
17. Hakulinen N, Tenkanen M, Rouvinen J (2000) Three-dimensional structure of the catalytic core of acetylxylan esterase from *Trichoderma reesei*: insights into the deacetylation mechanism. *J Struct Biol* 132(3): 180-190. [↑](#endnote-ref-18)
18. Hermoso J, et al. (2004) The crystal structure of feruloyl esterase A from *Aspergillus niger* suggests evolutive functional convergence in feruloyl esterase family. *J Mol Biol* 338(3): 495-506. [↑](#endnote-ref-19)
19. Raves ML, et al. (1997) Structure of acetylcholinesterase complexed with the nootropic alkaloid, (-)-huperzine A. *Nat Struct Biol* 4(1): 57-63. [↑](#endnote-ref-20)
20. Bencharit S, et al. (2006) Multisite promiscuity in the processing of endogenous substrates by human carboxylesterase 1. *J Mol Biol* 363(1): 201-214. [↑](#endnote-ref-21)
21. Yang L, Hill M, Wang M, Panjikar S, Stockigt J (2009) Structural basis and enzymatic mechanism of the biosynthesis of C9- from C10-monoterpenoid indole alkaloids. *Angew Chem Int Edit* 48(28): 5211-5213. [↑](#endnote-ref-22)
22. van den Berg B (2010) Crystal structure of a full-length autotransporter. *J Mol Biol* 396(3): 627-633. [↑](#endnote-ref-23)
23. Katona G, et al. (2002) X-ray structure of a serine protease acyl-enzyme complex at 0.95-A resolution. *J Biol Chem* 277(24): 21962-21970. [↑](#endnote-ref-24)
24. Kishi T, et al. (1999) Crystal structure of neuropsin, a hippocampal protease involved in kindling epileptogenesis. *J Biol Chem* 274(7): 4220-4224. [↑](#endnote-ref-25)
25. Bode W, et al. (1989) The refined 1.9 A crystal structure of human alpha-thrombin: interaction with D-Phe-Pro-Arg chloromethylketone and significance of the Tyr-Pro-Pro-Trp insertion segment. *EMBO J* 8(11): 3467-3475. [↑](#endnote-ref-26)
26. Gordon EJ, Mouz N, Duee E, Dideberg O (2000) The crystal structure of the penicillin-binding protein 2x from *Streptococcus pneumoniae* and its acyl-enzyme form: implication in drug resistance. *J Mol Biol* 299(2): 161-170. [↑](#endnote-ref-27)
27. Scheidig AJ, Hynes TR, Pelletier LA, Wells JA, Kossiakoff AA (1997) Crystal structures of bovine chymotrypsin and trypsin complexed to the inhibitor domain of Alzheimer's amyloid beta-protein precursor (APPI) and basic pancreatic trypsin inhibitor (BPTI): engineering of inhibitors with altered specificities. *Protein Sci* 6(9): 1806-1824. [↑](#endnote-ref-28)
28. Radisky ES, Kwan G, Karen Lu CJ, Koshland Jr DE (2004) Binding, proteolytic, and crystallographic analyses of mutations at the protease-inhibitor interface of the subtilisin BPN'/chymotrypsin inhibitor 2 complex. *Biochemistry* 43(43): 13848-13656. [↑](#endnote-ref-29)
29. Maynes JT, Cherney MM, Qasim MA, Laskowski Jr M, James MN (2005) Structure of the subtilisin Carlsberg-OMTKY3 complex reveals two different ovomucoid conformations. *Acta Crystallogr D* 61(Pt 5): 580-588. [↑](#endnote-ref-30)
30. McLuskey K, Paterson NG, Bland ND, Isaacs NW, Mottram JC (2010) Crystal structure of *Leishmania major* oligopeptidase B gives insight into the enzymatic properties of a trypanosomatid virulence factor. *J Biol Chem* 285(50): 39249-39259. [↑](#endnote-ref-31)
31. Liang L, et al. (2010) The crystal structures of two cuticle-degrading proteases from *Nematophagous* fungi and their contribution to infection against nematodes. *FASEB J* 24(5): 1391-1400. [↑](#endnote-ref-32)
32. Medrano FJ, et al. (1998) Structure of proline iminopeptidase from *Xanthomonas campestris* pv. citri: a prototype for the prolyl oligopeptidase family. *EMBO J* 17(1): 1-9. [↑](#endnote-ref-33)
33. McVey CE, Walsh MA, Dodson GG, Wilson KS, Brannigan JA (2001) Crystal structures of penicillin acylase enzyme-substrate complexes: structural insights into the catalytic mechanism. *J Mol Biol* 313(1): 139-150. [↑](#endnote-ref-34)
34. Line K, Isupov MN, Littlechild JA (2004) The crystal structure of a (-) gamma-lactamase from an *Aureobacterium* species reveals a tetrahedral intermediate in the active site. *J Mol Biol* 338(3): 519-532. [↑](#endnote-ref-35)
35. Labahn J, Neumann S, Buldt G, Kula MR, Granzin J (2002) An alternative mechanism for amidase signature enzymes. *J Mol Biol* 322(5): 1053-1064. [↑](#endnote-ref-36)
36. Silvaggi NR, Anderson JW, Brinsmade SR, Pratt RF, Kelly JA (2003) The crystal structure of phosphonate-inhibited D-Ala-D-Ala peptidase reveals an analogue of a tetrahedral transition state. *Biochemistry* 42(5): 1199-1208. [↑](#endnote-ref-37)
37. Goettig P, Groll M, Kin JS, Huber R, Brandstetter H (2002) Structures of the tricorn-interacting aminopeptidase F1 with different ligands explain its catalytic mechanism. *EMBO J* 21(20): 5343-5352. [↑](#endnote-ref-38)
38. Yoshimoto T, et al. (1999) Crystal structure of prolyl aminopeptidase from *Serratia marcescens*. *J Biochem* 126(3): 559-565. [↑](#endnote-ref-39)
39. Yasuhira K, et al. (2010) X-ray crystallographic analysis of the 6-aminohexanoate cyclic dimer hydrolase: catalytic mechanism and evolution of an enzyme responsible for nylon-6 byproduct degradation. *J Biol Chem* 285(2): 1239-1248. [↑](#endnote-ref-40)
40. Varshney NK, et al. (2012) Crystallization and X-ray structure analysis of a thermostable penicillin G acylase from *Alcaligenes faecalis*. *Acta Crystallogr F* 68(Pt 3): 273-277. [↑](#endnote-ref-41)
41. Mileni M, et al. (2010) X-ray crystallographic analysis of alpha-ketoheterocycle inhibitors bound to a humanized variant of fatty acid amide hydrolase. *J Med Chem* 53(1): 230-240. [↑](#endnote-ref-42)
42. DOI:10.2210/pdb3nwo/pdb [↑](#endnote-ref-43)
